# Recombinant oncolytic poliovirus, PVSRIPO, has potent cytotoxic and innate inflammatory effects, mediating therapy in human breast and prostate cancer xenograft models

**DOI:** 10.18632/oncotarget.12975

**Published:** 2016-10-28

**Authors:** Eda K. Holl, Michael C. Brown, David Boczkowski, Megan A. McNamara, Daniel J. George, Darell D. Bigner, Matthias Gromeier, Smita K. Nair

**Affiliations:** ^1^ Department of Surgery, Duke University School of Medicine, Durham, NC 27710, USA; ^2^ Department of Neurosurgery, Duke University School of Medicine, Durham, NC 27710, USA; ^3^ Department of Medicine, Duke University School of Medicine, Durham, NC 27710, USA; ^4^ Department of Pathology, Duke University School of Medicine, Durham, NC 27710, USA

**Keywords:** oncolytic poliovirus, PVSRIPO, inflammation, innate immunity

## Abstract

Intratumoral inoculation of viruses with tumor-selective cytotoxicity may induce cancer cell death and, thereby, shrink neoplastic lesions. It is unlikely, however, that viral tumor cell killing alone could produce meaningful, durable clinical responses, as clinically suitable ‘oncolytic’ viruses are severely attenuated and their spread and propagation are opposed by host immunity. Thus, a more propitious event in this context is the innate antiviral response to intratumoral virus administration, in particular for recruiting durable adaptive immune effector responses. It may represent a double-edged sword, as innate immune activation may eliminate infected tumor cells early, intercept viral spread and block any meaningful therapeutic response. The innate response to viral infection of tumors may be very different from that in non-malignant target tissues, owing to the unusual composition/tissue properties of tumor stroma. In this work, we report investigations of the innate immune response to the oncolytic poliovirus recombinant, PVSRIPO, in two mouse xenotransplantation models for breast and prostate cancer. Our observations indicate short-term virus persistence in infected tumors and virus recovery indicative of modest intratumoral propagation and persistence. Yet, a powerful innate inflammatory response coincided with chemokine induction and myeloid cell infiltration into tumors that was, interestingly, dominated by neutrophils. The combined effect of PVSRIPO tumor infection and the innate response it elicits was significant tumor regression in both models.

## INTRODUCTION

Prostate and breast cancers represent the most common malignancies in American men and women, respectively and are the second most common causes of cancer-related mortality in each group [1]. Despite the approval of new therapies in recent years, high risk locally advanced breast and prostate cancers (stage III) often recur after treatment [2–8], and metastatic disease remains incurable. Thus, novel therapies that prevent mortality associated with prostate and breast cancers are urgently needed.

Oncolytic viruses are replication competent, tumor-selective viruses that are capable of infecting and killing cancer cells [9]. Members of different viral families, including DNA and RNA viruses, have been investigated in numerous clinical trials [10, 11]. In October 2015, a genetically modified herpes simplex virus type 1 (talimogene laherparepvec; T-VEC, Amgen) was the first oncolytic virus to gain FDA approval for the local treatment of unresectable recurrent melanoma [11]. Indeed, based on encouraging pre-clinical data, several (~ 40) clinical trials are currently ongoing in multiple cancers using different DNA and RNA oncolytic virus strategies to assess oncolytic virus efficacy in the clinic [12].

A key feature of oncolytic virus antitumor efficacy is the ability to target and infect heterogeneous tumors, cause direct cytotoxic killing of infected tumor cells, and engender potent and durable secondary immune effector mechanisms against cancer cells. To elicit these effects consistently and successfully, an oncolytic virus needs to function in the context of an immunosuppressive tumor microenvironment, be non-cytopathogenic in normal cells, and maintain sufficient replication to impact tumor growth/inflammation despite anti-viral responses [9].

We have developed an oncolytic polio:rhinovirus recombinant, PVSRIPO, which is under investigation as an anti-cancer therapeutic in patients with recurrent glioblastoma, a notoriously treatment-refractory cancer. PVSRIPO has demonstrated remarkable durable complete clinical and radiographic responses in a proportion of these patients and was granted Breakthrough Therapy Designation by the FDA on May 10th, 2016. PVSRIPO is the type 1 (Sabin) live-attenuated poliovirus vaccine carrying a heterologous internal ribosomal entry site (IRES) of human rhinovirus type 2 (HRV2). The HRV2 IRES mediates neuronal incompetence of PVSRIPO, by forming a ribonucleoprotein complex that is incompatible with viral IRES-mediated translation specifically in neuron-lineage cells [13]. Thus, PVSRIPO is non-pathogenic by virtue of its inability to translate and propagate in the normal human CNS. PVSRIPO retains translation competence and cytotoxicity in neoplastic cells, which offer ideal conditions for viral IRES-mediated ribosome recruitment due to unhinged protein synthesis control in cancer [14, 15]. PVSRIPO enters cells via the poliovirus receptor, CD155, which is ectopically expressed in virtually all solid neoplasias [16–20], except possibly Burkitt lymphoma (where EBV infection interferes with CD155 upregulation [21]), including breast and prostate cancers [22, 23]. Therefore, PVSRIPO could have utility in the immunotherapy of breast and prostate cancers. As a first step towards this goal, our objective for this study was to assess the efficacy of PVSRIPO in mouse xenotransplantation models of breast and prostate cancer.

Poliovirus is an exclusively human pathogen (the virus only binds human/old world primate CD155; natural polio infection has only been reported in humans). Accordingly, it is appropriate to first assess PVSRIPO efficacy in mouse xenotransplantation models, despite their limitations in terms of modeling an intact immune response. Herein we present compelling evidence of PVSRIPO mediated antitumor activity in xenograft models of prostate and breast cancers. We demonstrate that tumor regression and antitumor efficacy of PVSRIPO produces potent innate immune activation and immune infiltration into the tumor. Our experiments uncovered an unexpected role of tumor-infiltrating neutrophils in the response to PVSRIPO therapy and provide a solid rationale towards further clinical development of PVSRIPO oncolytic immunotherapy against prostate and breast cancer.

## RESULTS

### PVSRIPO lyses and propagates in human SUM149 breast cancer and DU145 prostate cancer cells *in vitro*

**Figure 1:**
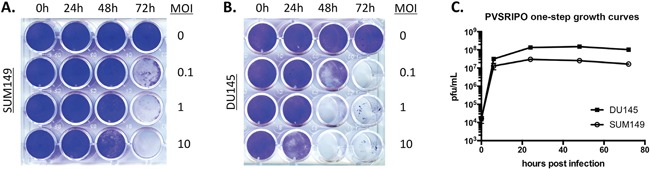
PVSRIPO effectively lyses SUM149 breast cancer cells and DU145 prostate cancer cells SUM149 **(A)** and DU145 **(B)** cells were seeded at a density of 5 × 10^4^ cells per well. Cells were exposed to various concentrations of PVSRIPO (MOI of 0, 0.1, 1 and 10 MOIs) for 0, 24, 48 and 72 hours. Cell lysis was assessed using crystal violet staining. (C) PVSRIPO propagation following infection (MOI of 10) of DU145 and SUM149 cells was assessed by plaque assay at the designated time points. Data are representative of at least 3 independent experiments; note that titers in **(C)** are from an assay distinct from (A) and (B).

The poliovirus receptor (CD155) is necessary and sufficient to mediate susceptibility to poliovirus in human cells and is expressed on the vast majority of solid cancer cell lines [24]. To test susceptibility of prostate and breast cancer cell lines to PVSRIPO, we measured tumor cell lysis after PVSRIPO infection using crystal violet to stain remaining live cells post infection. For this purpose, virus was added to tissue culture wells and infected cultures were stained at the indicated intervals with crystal violet. Cancer cells were completely lysed within 72 hrs (SUM149, Figure 1A) or 48 hrs (DU145, Figure 1B) at all multiplicities of infection (MOI) tested. We next assessed viral propagation (Figure 1C) over a 72 hr time period with a one-step growth curve assay. Total viral yield in DU145 cells was approximately 6-fold higher than that of SUM149, conceivably correlating with the difference in viral cytotoxicity between the two lines. Viral propagation peaked prior to complete cell lysis, possibly because viral egress occurs prior to cell lysis. Also, detachment of dead cells from substrate (resulting in loss of crystal violet stain) occurs much later than viral cell killing. The mechanism of poliovirus cell killing is poorly defined, due to the rapid progression of multiple highly cytotoxic events that shut down host protein synthesis, disrupt nucleo-cytoplasmic transport, remodel the cytoplasm, disintegrate intracellular vesicular transport and destroy the integrity of the plasma membrane [25, 26]. These data demonstrate that PVSRIPO propagates in and has potent cytotoxic effects in SUM149/DU145 cells *in vitro* within hours and may exhibit similar effects *in vivo*.

### A single intratumoral injection of PVSRIPO is effective at shrinking SUM149 breast cancer and DU145 prostate cancer tumors *in vivo*

**Figure 2:**
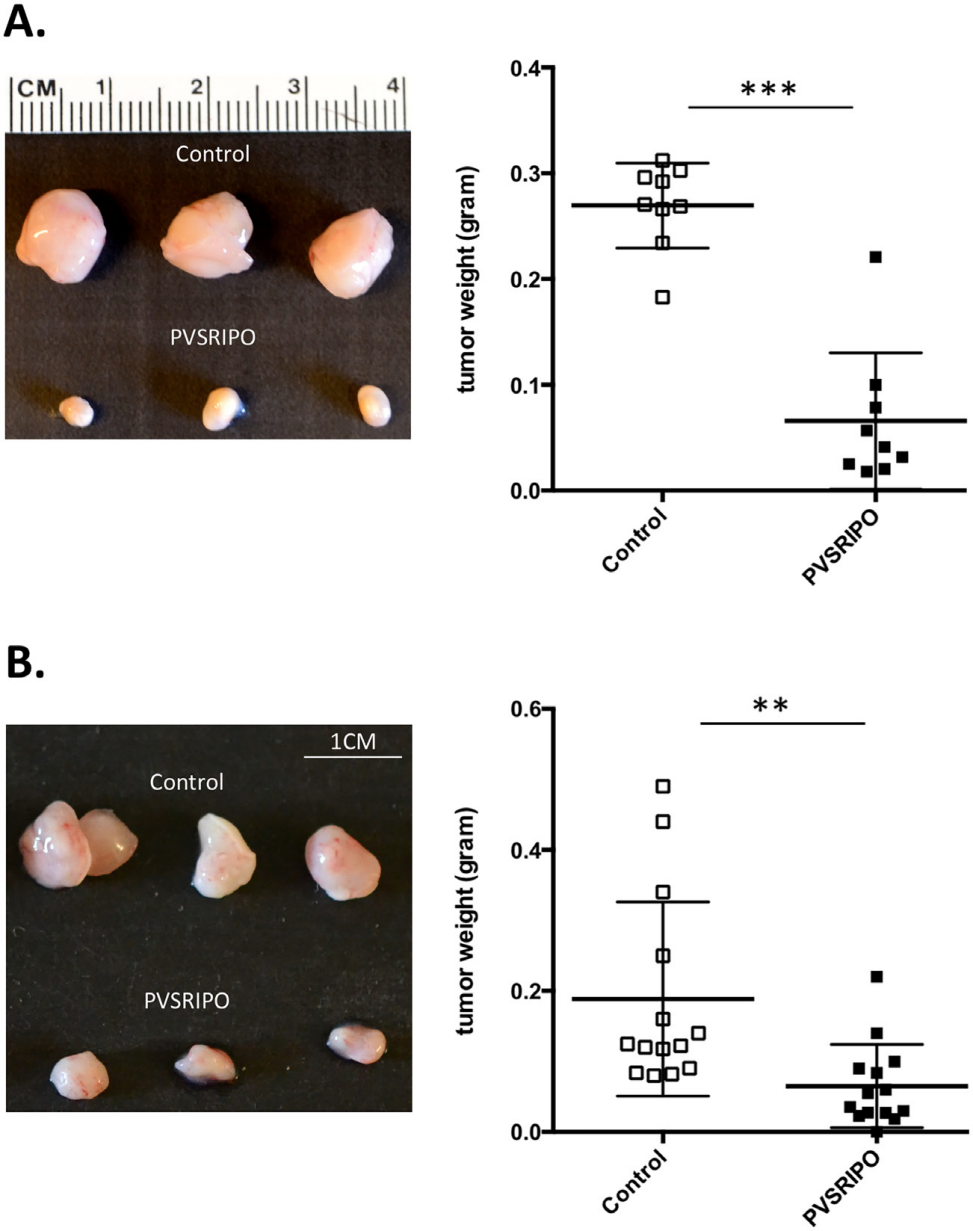
Single intratumoral administration of PVSRIPO results in tumor regression in SUM149 and DU145 xenografts SUM149 and DU145 cells were implanted in athymic nu/nu mice. Tumors were injected with 10^8^ pfu of PVSRIPO when they reached 150-200 mm^3^. Tumors were collected at 7 days post injection and size and weight were assessed. **(A)** SUM149 tumor data are representative of five different experiments and a total of 25 mice per group. Graph represents tumor weights (grams) from one representative experiment. **(B)** DU145 tumor data are representative of five different experiments and a total of 25 mice per group. Graph represents tumor weights (grams) from one representative experiment. ***p<0.001.

*In vitro* data in Figure 1 demonstrate that PVSRIPO efficiently lyses DU145 and SUM149 cells. To determine whether this antitumor effect translated to or is predictive in an *in vivo* setting, we tested PVSRIPO in two different rodent tumor xenotransplantation models: orthotopic SUM149 breast cancer and subcutaneous DU145 prostate cancer. Once tumors reached a volume of 150-200 mm^3^, a single dose of PVRSIPO (10^8^ pfu) was injected intratumorally. Tumor growth was monitored and mice were sacrificed when tumors reached 2000 mm^3^ or mice became moribund. A single dose of PVSRIPO was sufficient to substantially delay tumor growth by day-7 post injection, as measured by tumor weight, with ~7-fold (SUM149) and ~3-fold (DU145) decline in weight as compared to mock-treated tumors (Figure 2A, 2B). Together these results demonstrate that PVSRIPO's antitumor cytotoxic effect in SUM149/DU145 cells may qualify it as a potential therapeutic agent for treatment of patients with breast and prostate cancer.

### PVSRIPO induces innate immune gene expression in SUM149 breast cancer and DU145 prostate cancer tumors

**Figure 3:**
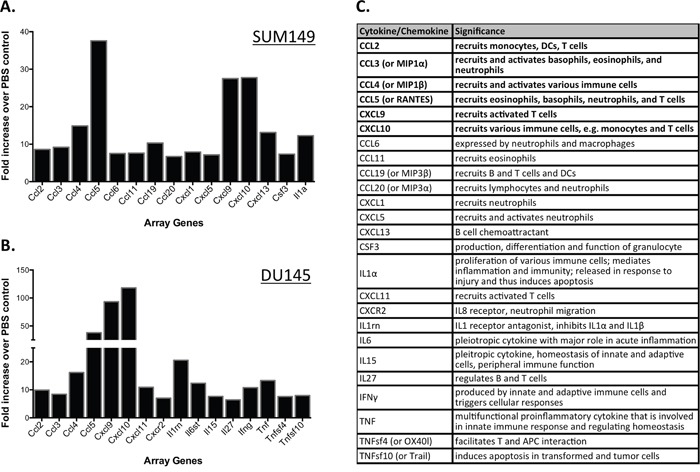
PVSRIPO upregulates transcripts encoding for innate immune-related gene products in SUM149 and DU145 xenografts SUM149 **(A)** and DU145 **(B)** cells were implanted in athymic nu/nu mice. Tumors were injected with 10^8^ pfu of PVSRIPO when they reached 150-200 mm^3^. Tumors were collected at 24 hours post injection and innate immune-related transcript abundance was assessed. Data are representative of two different experiments and a total of 6 mice per group. Only changes of ≥8-fold are reported. **(C)** Cytokines/chemokines represented in (A, B) and their function in inflammation; cytokines shown in bold were induced in both tumors. Information about cytokine significance was obtained from Uniprot [43] and NCBI databases.

PVSRIPO efficiently lysed tumor cells *in vitro* and reduced tumor burden in mouse xenograft tumor models. The limited extent of PVSRIPO propagation in tumors (see below), in particular when compared to the rampant growth of neurovirulent polioviruses in the spinal cord of infected *CD155*-transgenic mice [13], indicates that anti-tumor effects may not be primarily due to direct viral cytotoxicity. Rather, much evidence suggests that PVSRIPO elicits host inflammatory responses that may contribute to tumor rejection through immunologic mechanisms [9]. In the context of cancer immunotherapy, the innate antiviral inflammatory response to PVSRIPO could enable the production of such antitumor immunity directly (TNF-α and NK-cell mediated killing) and indirectly (antitumor T cell and antibody responses). Therefore, we investigated if the host innate immune system is engaged following intratumoral PVSRIPO administration. To identify early, immediate inflammatory effects of PVSRIPO treatment on the tumor microenvironment/stroma, SUM149 and DU145 tumors were harvested and mRNA was extracted 24 hours post-PVSRIPO or PBS administration. mRNA from each mouse was processed individually and analyzed using gene arrays for murine pro-inflammatory cytokines. To highlight the prominent cytokines/chemokines induced by viral oncolysis, only changes greater than 8-fold are reported (Figure 3A, 3B). Our data revealed significantly increased expression of pro-inflammatory chemokines and cytokines at 24 hrs in PVSRIPO-treated tumors as compared to PBS-treated controls. A table outlining the significance of each cytokine is shown in Figure 3C. CCL-2, 3, 4, and CXCL-9 and 10 were induced in both SUM149 and DU145 tumors; which collectively, may enable the recruitment of immune cells into the tumor (Figure 3). Of specific significance, cytokines that recruit/activate granulocytes or are produced by granulocytes (including basophils, eosinophils, and neutrophils) were induced in both contexts. These include CCL5, CCL6, CCL11, CCL20, CXCL5, CSF3, and CXCR2 (Figure 3C). Of note, the chemokines CCL5 and CXCL10 are involved in T cell recruitment [27, 28]. Intriguingly, the response in each tumor model demonstrates differences in pattern and intensity. This may be due to cell type-specific differences in anti-viral/pro-inflammatory responsiveness in SUM149 and DU145 tumors. Fast, robust chemokine and cytokine induction in tumors following intratumoral PVSRIPO administration may indicate involvement of the innate immune system in PVSRIPO-mediated antitumor responses.

### PVSRIPO recruits innate immune cells into SUM149 breast cancer and DU145 prostate cancer tumors

**Figure 4:**
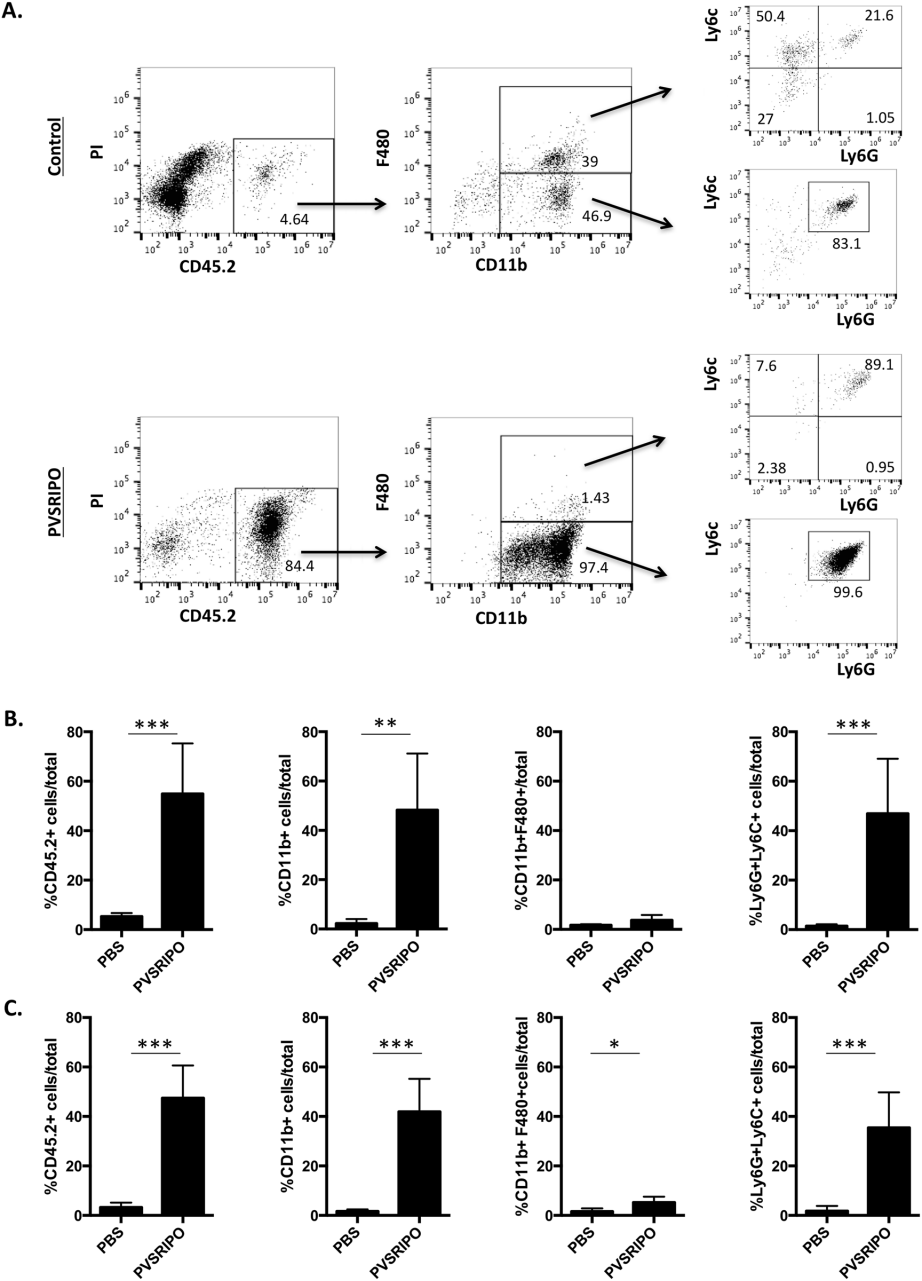
PVSRIPO recruits innate immune cells into SUM149 and DU145 xenografts **(A)** SUM149 and DU145 cells were implanted in athymic nu/nu mice. Tumors were injected with 10^8^ pfu of PVSRIPO when they reached 150-200 mm^3^. Tumors were collected at 48 hours post injection and innate immune cell infiltration was assessed by flow cytometry (CD45.2, CD11b, F480, Ly6C, Ly6G, B220, CD335). The numbers reflect percentage of cells in the gated population (boxed area) in the preceding flow quadrant as indicated. Data are representative of three different experiments and a total of 15 mice per group. **(B)** Tumor infiltrating immune cell percentages from one representative experiment (n=5) in the SUM149 breast tumor model are shown. ***p<0.001. **(C)** Tumor infiltrating immune cell percentages from one representative experiment (n=5) in the DU145 prostate tumor model are shown. *p<0.05, **p<0.01, ***p<0.001.

Rapid induction of chemokine and cytokine production after PVSRIPO treatment suggested that innate immune cell infiltration may ensue. To further elucidate the immune response elicited by PVSRIPO in SUM149 and DU145 tumors, we assessed the percentage of tumor-infiltrating immune cells at 48 hrs post PVSRIPO treatment. Flow cytometry analysis of the immune cells revealed significantly increased infiltration of hematopoietic origin cells in the treated animals compared to untreated controls (Figure 4A). The majority of the immune cells present were CD11b+. Further analysis of these populations revealed that cells obtained from PVSRIPO treated tumors were almost entirely CD11b+Ly6C+Ly6G+, consistent with a neutrophil phenotype. Neutrophils represent 1.8% of total cells in the tumors of mock (PBS) treated mice versus a remarkable 81.8% in PVSRIPO-treated tumors (Figure 4A). To illustrate this phenomenon further, we show consolidated data from SUM149 tumors and DU145 tumors of immune cell infiltrates into the tumor. Neutrophils represent the majority of the infiltrating cells into the tumors (right panel depicting CD11b+Ly6C+Ly6G+ in Figure 4B and 4C). Collectively, these data indicate that PVSRIPO therapy induces a rapid influx of hematopoietic cells that are predominately neutrophils, which are crucial factors for regulating inflammation and the development of adaptive immune responses.

**Figure 5:**
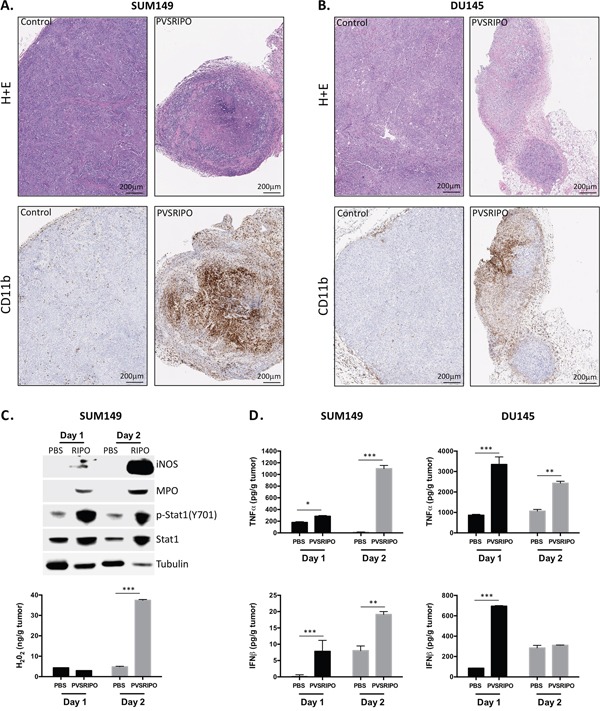
Innate immune cell infiltration after PVSRIPO is associated with signs of cell-mediated tumor cytotoxicity SUM149 and DU145 cells were implanted in athymic nu/nu mice. Tumors were injected with 10^8^ pfu of PVSRIPO when they reached 150-200 mm^3^. Tumors were collected at 7 days post injection and immune cell recruitment was assessed by histology (H&E) and immunohistochemistry (CD11b). Data are representative of two different experiments and a total of 10 mice per group. SUM149 **(A)** and DU145 **(B)** tumors demonstrate significantly increased infiltration of CD11b+ immune cells. **(C, D)** SUM149 tumor homogenates from mock (PBS) or PVSRIPO-treated mice were tested (C, top) for markers of neutrophil and innate immune cell inflammation by immunoblot; (C, bottom) for the presence of H_2_O_2_; (D) for the presence of TNF-α and IFN-β by ELISA.

Next we analyzed neutrophil distribution within tumors 7 days post PVSRIPO administration. Tumors were harvested from mice and tumor sections were analyzed by H&E and immunohistochemistry (IHC) for the presence of tumor-infiltrating CD11b+ immune cells. As indicated in Figure 5, PVSRIPO-treated tumors in both SUM149 (Figure 5A) and DU145 (Figure 5B) groups revealed increased numbers of infiltrating immune cells. This observation is remarkable given the paucity of stromal CD11b+ immune cells present in PBS-treated tumors (Figure 5). These data reveal significant immune cell activity in response to PVSRIPO-mediated tumor cell infection and destruction that may contribute to therapy.

### Intratumoral PVSRIPO-induced inflammation favors innate anti-tumor activity

Infiltrating neutrophils and other immune populations responding to localized, virus-induced inflammation may also function by killing cancer cells directly. This mode of action could be critical in bolstering anti-tumor immunity while also directly restricting cancer cell growth. Similar to macrophages, tumor-associated neutrophils paradoxically enhance and restrict tumor growth in a context-specific manner [29, 30]. During infection with a pathogen, however, it is likely that neutrophils engage inflammatory and cytotoxic processes that are unfavorable to tumor (and viral) growth. Mechanisms by which neutrophils and other CD11b+ cell types may kill cancer cells include TNF-α secretion/TRAIL mediated killing, Reactive Oxygen Species (ROS) including hydrogen peroxide (H_2_O_2_) and nitric oxide (NO), and possibly contact-dependent killing similar to that of NK cells [30]. To address whether these mechanisms are active following PVSRIPO therapy, tumor homogenates were tested by immunoblot and ELISA for evidence of antitumor/pro-inflammatory innate activation at 24 and 48 hrs post treatment (Figure 5C, 5D). Immunoblot of SUM149 tumor lysates revealed that PVSRIPO treatment increased intratumoral expression of the following: 1] iNOS, an enzyme responsible for producing NO; 2] myeloperoxidase, an enzyme predominately expressed by neutrophils that catalyzes the production of cytotoxic free radicals; 3] p-Stat1 (Y701), phosphorylated downstream of both type-I and -II IFN signaling; and 4] Stat1, which is induced by its own phosphorylation (Figure 5C). Consistent with increased neutrophil activity, higher H_2_O_2_ concentration was also detected in SUM149 tumor lysate supernatant following PVSRIPO treatment (Figure 5C). Lastly, tumor homogenate supernatants from both SUM149 and DU145 cells were tested by ELISA for TNF-α and IFN-β, both of which could either directly or indirectly have tumoricidal effects. TNF-α was produced in both tumor contexts, but consistent with RNA expression in Figure 3, TNF-α production in DU145 tumors was more pronounced earlier on (Figure 5D). Induction of type-I interferon was also observed, similarly with DU145 tumors having much more robust IFN-β responses at 24 hrs than the SUM149 tumors (Figure 5D). Altogether, these data indicate that PVSRIPO intratumoral therapy not only leads to infiltration of neutrophils, but also induces multifaceted pro-inflammatory, potentially anti-tumor, innate immune cell-mediated events.

### A single intratumoral dose of PVSRIPO effectively shrinks SUM149 breast cancer and DU145 prostate cancer tumors and increases survival

**Figure 6:**
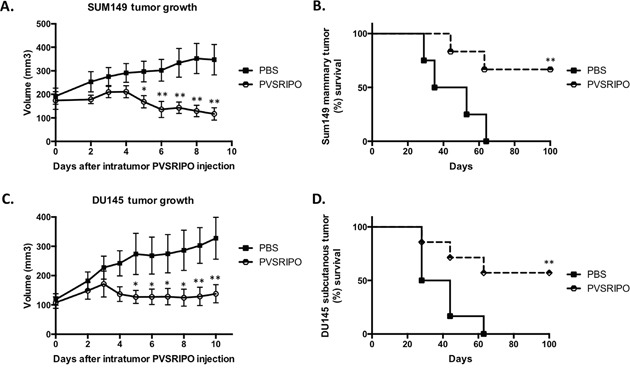
Single intratumoral administration of PVSRIPO in SUM149 and DU145 xenografts results in tumor regression and survival benefit SUM149 and DU145 cells were implanted in athymic nu/nu mice. Tumors were injected with 10^8^ pfu of PVSRIPO when they reached 150-200 mm^3^. Tumor growth was monitored daily and mice were sacrificed when tumors reached 2000 mm^3^. Data are representative of 3 different experiments (n=30). SUM149 **(A, B)** and DU145 **(C, D)** tumor growth 7 days post PVRIPO injection and overall survival up to 100 days.

Tumor elimination/shrinking is ultimately required for the efficacy of anti-tumor therapies. As part of our evaluation of *in vivo* anti-tumor effects of PVSRIPO we measured tumor volume in mice bearing SUM149 and DU145 tumors. A single administration of PVSRIPO into the SUM149 and DU145 xenografts resulted in suppressed tumor growth (Figure 6A, 6C). Moreover, the overall survival rates in these mice were improved as compared to the PBS-treated counterparts (Figure 6B, 6D). A cohort of the treated mice in both cancer models achieved complete tumor regression and extended overall survival. As expected, none of the treated mice experienced adverse side effects following PVSRIPO treatment (wild-type mice are resistant to poliovirus infection). Together these data demonstrate that single, intratumoral administration of PVSRIPO, and the potent innate inflammatory response it generates, result in tumor regression. As these responses represent universal mechanisms of the innate anti-viral defense, it is not surprising that they may occur in tumor models originating from different tumor histologies.

### PVSRIPO persistence in SUM149 breast tumors and DU145 prostate tumors following single, intratumoral injection

**Figure 7:**
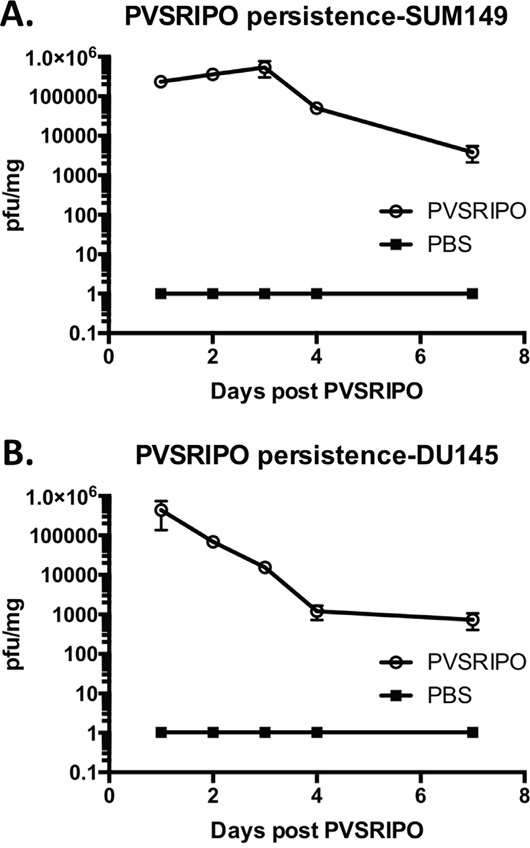
PVSRIPO persistence in SUM149 and DU145 xenografts following single intratumoral injection SUM149 **(A)** and DU145 **(B)** cells were implanted in athymic nu/nu mice. Tumors were injected with 10^8^ pfu of PVSRIPO when they reached 150-200 mm^3^. Tumors were isolated at 1, 2, 3, 4 and 7 days post virus inoculation. Pfu per mg of tumor were determined by plaque assay and plotted; data are representative of two independent experiments (n=30).

As a +strand RNA virus unable to chronically persist, except in rare instances of persistence in the enteric tract of patients with severe inherited/acquired immune deficiencies [31], PVSRIPO presence and spread in tumors likely is limited. To test the incidence of PVSRIPO persistence in breast and prostate xenotransplantation tissues and to correlate viral replication with inflammation following intratumoral administration, we measured viral presence for a period of 1-week post injection into tumors. The presence of PVSRIPO was detected in both SUM149 and DU145 tumors; however, tumor-associated titers did not increase over time in SUM149 tumors (Figure 7A) and declined throughout the observation period in DU145 tumors (Figure 7B). These data are indicative of low-level viral propagation/spread, which is in agreement with our published observation wherein virus recovery from xenografts 10 days post intratumor PVSRIPO injection was negligible in the range of 2-12 pfu/mg of tumor [32]. Our data suggests that this low-level intratumoral presence of PVSRIPO provides a favorable stimulus for immune cell infiltration into the tumor.

Innate cell-mediated killing of infected tumor cells and antiviral responses likely explain the stifling of viral propagation over time in both models. Indeed, more potent IFN-β and TNF-α responses were observed soon after treatment in DU145 tumors compared to SUM149 tumors (Figure 5C), possibly explaining less impressive viral replication/persistence in the DU145 context (Figure 7). In addition, the pattern and intensity of the immune response against the tumor or the responsiveness of the tumor cells to innate immune responses (such as sensitivity to TNF-α or antiviral responses) may be contributing factors. Altogether, our observations suggest that low-level PVSRIPO propagation in breast and prostate cancers, combined with the powerful innate inflammatory response it engenders, can mediate control of neoplastic lesions.

## DISCUSSION

Despite recent therapeutic advances, metastatic castrate-resistant prostate cancer and metastatic breast cancer remain incurable. Anti-cancer therapies that engage the immune system and promote durable systemic immune surveillance may be most adept at achieving meaningful disease control. In this work, we present a strategy that targets and damages malignant cells and, in the process, provides suitable recognition patterns for engendering anti-tumor immune surveillance.

PVSRIPO has produced remarkable clinical and radiographic responses in patients with recurrent GBM [9]. The primary objective of this study was to investigate if PVSRIPO immunotherapy is feasible in prostate and breast cancers. Our findings provide a rational basis for initiating clinical translation of PVSRIPO for the treatment of breast and prostate cancers. Furthermore, our studies define mechanistic principles that may underpin PVSRIPO oncolytic immunotherapy, in particular with regard to the role of innate pro-inflammatory activation.

Oncolytic immunotherapy relies on a virus' ability to target tumor cells, enter and damage or kill tumor cells and, most importantly, recruit immune cells to the tumor site and provide a pro-inflammatory stimulus within the deeply immune-suppressive microenvironment of tumor stroma. While syngeneic, immunocompetent mouse models are required to fully assess the immunotherapeutic aspects of any modality, athymic nude (nu/nu) mouse xenograft models enable the study of human-derived tumors. However, tumor xenotransplantation models in athymic nude mice have a number of limitations that must be considered, given their lack of T cells. Additionally, these xenograft models suffer from 1) unknown effectiveness of human IFN in a mouse setting; 2) uncertain effects of mouse IFN on human xenografts; 3) an inability of murine cytokines to intercept the infection, favoring virus persistence; and 4) inefficient killing of human tumor cells such as SUM149 and DU145 by murine immune cells.

Nude mice have intact innate immune systems that facilitate the study of key innate immune events critical to immunotherapy outcomes. Polioviruses cannot infect murine tumor models and, therefore, syngeneic immune-competent models require engineering of mouse tumor cell lines to express CD155. Lastly, the heterologous HRV2 IRES in PVSRIPO is not fully functional in murine cells [33]. Thus, for use in murine tumor models, PVSRIPO must be adapted for mouse competence. While we are proceeding with generating appropriate syngeneic models to study PVSRIPO in immune-competent hosts, it is equally important to investigate PVSRIPO oncolytic immunotherapy in human cancers. The identification of key effectors of the innate immune response to PVSRIPO oncolysis enables targeted follow-up studies in immunocompetent mouse models.

Earlier studies of PVSRIPO in glioma xenotransplantation models revealed histologic evidence of immune infiltration and tumor regress [24, 32, 34]. The innate immune mechanisms involved, however, remained unexplored. DU145 and SUM149 tumors also responded to PVSRIPO, evident by rapid tumor regression compared to their control (PBS)-treated counterparts. We demonstrated that potent induction of an array of chemokines and chemokine receptors within the tumor microenvironment likely explain the recruitment of innate immune cells, including neutrophils, observed as early as 2 days post-treatment and persisting to 7 days post-treatment. Notably, CCL5 and CXCL10, chemokines responsible for T cell recruitment were induced [27, 28]. In future investigations we will assess the importance of T cell-recruiting chemokines in PVSRIPO oncolytic immunotherapy in immunocompetent mouse models of cancer.

Intriguingly, signs of neutrophil-mediated cytotoxicity were observed in PVSRIPO-treated SUM149 and DU145 tumors. This included TNF-α production, the synthesis of iNOS and MPO, and also the emergence of H_2_O_2_ within the tumor. The role of neutrophils in PVSRIPO oncolytic immunotherapy likely is complex and multifaceted. Direct tumoricidal effects may restrict intratumoral virus propagation and spread. Conversely, neutrophil/innate cell-mediated inflammation and tumor cell killing may be required for further anti-tumor immune cell recruitment and activation. For example, active neutrophils can regulate the NK-, T-, and B cell function through multiple mechanisms, many of which have only recently become appreciated [35]. Just as important may be type 1 interferon induction by PVSRIPO, a crucial part in the transition from innate to adaptive immune responses [36, 37]. Thus, our data point to the possibility of prolonged anti-tumor functions for infiltrating innate immune cells that extend far beyond initial PVSRIPO infection.

Collectively, our data support the notion that PVSRIPO functions through 1) direct viral cancer cell toxicity that is inextricably linked to 2) an innate and adaptive immune response that not only directs elimination of virus infected cells/the virus itself, but also against uninfected cancer cells [38, 39]. Future mechanistic investigations, pending successful development of immunocompetent murine cancer models, will further define the influence of early PVSRIPO:tumor host relations and neutrophil/innate immune cell recruitment and activation on adaptive, anti-tumor immunity. We hypothesize that the immune response generated through viral cytotoxicity represents the decisive factor in achieving therapeutic success. Most importantly, we will use the IND-enabling studies described in this manuscript to pursue clinical studies in breast and prostate cancer.

## MATERIALS AND METHODS

### Mice

Male and female BALB/c nude (nu/nu) mice were purchased from Duke University animal breeding facility. All animal experiments were performed under approved Duke University animal use protocol.

### Cell lines and virus

DU145 is a human prostate tumor cell line which was derived from metastatic site (brain) and was purchased through ATCC (ATCC^®^ HTB-81™; Manassas, VA). SUM149 is a human breast cancer cell line that was derived from a primary inflammatory ductal carcinoma of the breast, from a woman with locally advanced inflammatory breast cancer (IBC). SUM149 cells were a generous gift of Neil Spector (Duke University School of Medicine, Durham, NC). DU145 cells were grown in 10% FBS in DMEM (Invitrogen, Carlsbad, CA). SUM149 cells were grown in 10% FBS in Ham's DMEM-F12 medium (Lonza, Basel, Switzerland). All cell testing revealed no Mycoplasma contamination. HeLa cells were grown in DMEM supplemented with 10% FBS. PVSRIPO was grown in HeLa cells as previously described [40] and purified using a 0.45 μM syringe filter followed by concentration and filtration with a 100 KDa filter (Millipore, Billerica, MA).

### *In vivo* mouse xenograft tumor models

DU145 cells (2 × 10^6^ in 100 μl PBS) were injected subcutaneously into the right flank of nude mice. SUM149 cells (2 × 10^6^ in 100 μl PBS) were injected in the left abdominal mammary fatpad #4, under the nipple of nude mice. When tumors reached 6-8 mm in diameter they were injected once with PVSRIPO (10^8^ plaque forming units (pfu) in 20 μl) or PBS as a control. PBS was chosen as a control (as opposed to UV inactivated virus [19, 41]) because clinical lots and laboratory preparations of PVSRIPO contain more than 20-fold more non-infectious virus than infectious virus. Thus, we wanted to test combined effect of both infectious and non-infectious PVSRIPO and did not confine our focus to the effects of viral oncolysis and replication alone. Tumor measurements were taken daily and tumor volume was calculated as [length × (width)^2^]/2 and expressed as volume±SEM. Animals were euthanized when tumor volume exceeded 2000 mm^3^ or when mice became moribund.

### Flow cytometry

Tumors were isolated from mice 48 hours post-intratumor PVSRIPO injections. Tumors were gently minced and incubated for 30 minutes in RPMI-1640 containing 1.67 Wunsch units/ml liberase^TM^ (Roche, Basel, Switzerland) and 1 mg/ml DNase (Roche, Basel, Switzerland). Cells were filtered twice through 70 μm mesh filters and incubated with anti-mouse CD16/CD32 Fc receptor block. Cells were then stained with anti- CD335-FITC, -CD11b-PE, -CD11c-APC, CD45.2-KromeOrange, Ly6C-FITC, Ly6G-PE, B220-APC-Cy7, CD80-PECy7, CD86-PE, and F480-PE-Cy7 (Biolegend, San Diego, CA) antibodies and appropriate isotype controls for 20 minutes on ice. Data were acquired on a CytoFlex flow cytometer (Beckman Coulter, Miami, FL) and analyzed using FlowJo (Tree Star, Inc, Ashland, OR).

### *In vitro* and *in vivo* viral titers

For *in vitro* viral titer assessments, 1 × 10^6^ SUM149 or DU145 cells in 35mm dishes were infected with PVSRIPO (MOI 10). Following the virus attachment step (30 minutes at 37°C) cells were washed 3 times with serum free DMEM (Invitrogen, Carlsbad, CA) to begin time course experiments. Dishes were immediately frozen at the designated time points and analyzed by plaque assay to determine viral titers as previously described [42]. For *in vivo* tumor viral titers, tumors were harvested at designated intervals and frozen at −80°C until all samples were collected. Tumors were thawed, weighed, and homogenized in 1 ml PBS. The tumor homogenate was then tested by plaque assays as described above.

### ELISA, H_2_O_2_ measurement, and immunoblot

Tumor homogenate was prepared for *in vivo* viral titer assessment as described above. The homogenate supernatant was used for ELISA and western blot assay. TNF-α (eBioscience, San Diego, CA) and IFN-β (PBL Assay Science, Township, NJ) ELISA was performed according to manufacturer's instructions. H_2_O_2_ measurement was performed using a hydrogen peroxide chemiluminescent kit (Enzo Life Sciences, Farmingdale, NY) following manufacturer's instructions. ELISA and H_2_O_2_ data were corrected for tumor weight by dividing concentration by total tumor weight. Western blot was performed as previously described [15]. Briefly, 4x LDS buffer (Invitrogen, Carlsbad, CA) containing β-mercaptoethanol (Sigma-Aldrich, St. Louis, MO) was added to 1x and 5% (vol/vol) concentrations, respectively and gel electrophoresis using the Novex (Invitrogen, Carlsbad, CA) western blotting system was performed. A Licor Odyssey Fc imager (Licor, Lincoln, NE) was used to image immunoblots. Antibodies used for immunoblot recognized Nitric oxide synthase (NOS), Stat1-p (Y701), Stat1 (Cell Signaling Technologies, Danvers, MA); Tubulin (Sigma-Aldrich, St. Louis, MO); and myeloperoxidase (MPO; R&D Biosystems, Minneapolis, MN).

### Gene arrays

Tumors were isolated from mice 24 hours post-intratumor PVSRIPO injections. All tumors were homogenized for 5 minutes at room temperature using a Bullet Blender homogenizer (Next Advance, Averill Park, NY). RNA was isolated from tumor lysates using an RNA Isolation Kit (Qiagen, Hilden, Germany). cDNA was synthesized using an RT^2^ first strand kit (Qiagen, Hilden, Germany) and innate immune receptor expression was assessed using an RT^2^ profiler PCR array mouse inflammatory cytokines and receptors kit (Qiagen, Hilden, Germany). Data were analyzed using an online software program provided by Qiagen (Hilden, Germany). All changes greater than 8-fold were considered significant.

### Histology

DU145 and SUM149 tumors were isolated from mice 7 days post-intratumor PVSRIPO injections. Tumors were fixed in 10% formalin and paraffin embedded. Slides were processed from tumors and used for H&E staining and IHC staining for CD11b-expressing cells (Histowiz, New York, NY).

### Crystal violet stain

Crystal violet assay was performed by seeding 5×10^4^ tumor cells/well in a 24-well plate. Cells were mock-infected or infected with PVSRIPO at the designated multiplicity of infection (MOI), starting at 72-hour time point and proceeding with virus additions daily for 3 days; such that all the cells were analyzed at the same time. To stain living cells, crystal violet solution (1% crystal violet, 1% glutaraldehyde, and 50% methanol) was added directly to the cell medium, plates were rocked at room temperature for 1 hour, the stain was discarded and plates were washed in water.

### Statistical analysis

Data were analyzed using GraphPad Prism software. Statistical analysis was conducted using a 2-tailed unpaired Student t test, one-way ANOVA followed by Tukey multiple comparison test or non-parametric Mann-Whitney U test. P<0.05 was considered statistically significant. Survival curves were plotted using the Kaplan-Meier method (log-rank test).
